# One-Pot Tandem Alcoholysis-Hydrogenation of Polylactic Acid to 1,2-Propanediol

**DOI:** 10.3390/polym15020413

**Published:** 2023-01-12

**Authors:** Jialin Xu, Kuo Zhou, Linlin Qin, Zaiming Tan, Shijing Huang, Peigao Duan, Shimin Kang

**Affiliations:** 1 Engineering Research Center of None-Food Biomass Efficient Pyrolysis and Utilization Technology of Guangdong Higher Education Institutes, Guangdong Provincial Key Laboratory of Distributed Energy Systems, Dongguan University of Technology, Dongguan 523808, China; 2 School of Chemical Engineering and Technology, Xi’an Jiaotong University, Xi’an 710049, China; 3 Department of Chemistry, Lishui University, Lishui 323000, China

**Keywords:** polylactic acid, alcoholysis, hydrogenation, 1,2-propanediol, PLA straws

## Abstract

The chemical recycling of end-of-life polylactic acid (PLA) plays roles in mitigating environmental pressure and developing circular economy. In this regard, one-pot tandem alcoholysis and hydrogenation of PLA was carried out to produce 1,2-propanediol, which is a bulk chemical in polymer chemistry. In more detail, the commercially available Raney Co was employed as the catalyst, and transformation was conducted in ethanol, which acted as nucleophilic reagent and solvent. Single-factor analysis and Box–Behnken design were used to optimize the reaction conditions. Under the optimized condition, three kinds of PLA materials were subjected to degradation. Additionally, 74.8 ± 5.5%, 76.5 ± 6.2%, and 71.4 ± 5.7% of 1,2-propanediol was yielded from PLA powder, particle, and straws, respectively, which provided a recycle protocol to convert polylactic acid waste into value-added chemicals.

## 1. Introduction

Polylactic acid (PLA) is an environmental-friendly alternative to petroleum-derived plastic due to biodegradability and biocompatibility [1], which can be used in the following sectors: packaging, agriculture, textiles, clinical sector, and others [2]. In 2015, the global production of PLA was around 200 kt [3], and it is expected to reach 560 kt in 2025 [4], with an increase of 180% over the past decade. Thus, its tremendous growth in single-use products has led to serious social problems, e.g., economical inefficiency, excess wastes, and associated environmental concerns [5]. Although PLA is biodegradable, it takes decades to completely degrade in landfill [6], and 60–100 days are also needed under elevated temperature (50 ± 5 °C) in composting conditions [7]. These two routine degradation methods are not recommended choices since no material is recovered. In this regard, the chemical end-of-life scenario of short-life PLA is essential to determine its sustainability and circular economy, where monomer regeneration occurs, or value-added chemicals are produced. Chemical degradation of PLA generally includes hydrolysis, pyrolysis, alcoholysis, and ammonolysis [8]. A serious of products were derived from the above recycling options, such as lactic acid [9,10], lactide [11,12], lactate ester [13,14], biofuel [15,16], and others [17]. Hydrolysis occurs through random scission of the ester bond around 200 °C and the monomer of lactic acid is formed, which can further convert into new PLA and a variety of chemicals [10]. Pyrolysis takes place via intramolecular transesterification of PLA and results in lactide over the range of 200–500 °C [11,12]. Alcoholysis allows the formation of lactate esters, known as green solvents, and they can be transformed into lactide, resulting in circular economy [13,14]. Ammonolysis depolymerizes PLA into alanine, which also expands the application of PLA wastes [17].

In addition, PLA was found as a potential feedstock to produce 1,2-propanediol (1,2-PDO) as well [18,19], a bulk commodity chemical, which is extensively used as antifreeze, monomer for production of unsaturated polyester resins and biodegradable plastic, and additive in drug, food, and cosmetics [20]. Commonly used 1,2-PDO is obtained from propylene, which is primarily combined with ethylbenzene and chlorine to form propylene oxide, and then yield 1,2-PDO via hydration [21]. Regarding the depletion of fossil fuel and the environmental concern, there has been increased demand for bio-based processes using renewable feedstocks and waste streams, such as glycerol [22,23], glucose [24], cellulose [25], lactic acid [26,27], etc. (Table S1). Additionally, selective hydrogenolysis of glycerol is the most common renewable route for producing commercial 1,2-PDO, as glycerol is a low valued byproduct of biodiesel [28]. However, excess H_2_ and high energy input are necessary in the process, and byproducts (e.g., the acetol intermediate, ethylene glycol, propanol) are derived via further hydrogenolysis of glycerol [29]. Therefore, it provides a strong motivation to operationalize a new route for green and efficient production of 1,2-propanediol. 

In recent years, reductive depolymerization of PLA has emerged as an efficient alternative methodology to recover 1,2-PDO, employing ruthenium- [30,31], copper- [18], or zinc-based [32] catalysts, and H_2_ or silane as the reducing agent (Table S1). These recycling concepts from PLA to 1,2-PDO can not only greatly diminish the environmental concerns of PLA wastes, but also offer an attractive and renewable approach to 1,2-PDO. Nevertheless, one of the disadvantages of reported methods is the use of toxic, noble metal-based, and homogeneous catalysts. Moreover, expensive and not eco-friendly reagents should be avoided, such as toluene [32], 1,4-dioxane [31], THF, and anisole [19] (Table S1). In this regard, for sustainable industrial application of depolymerization PLA, it is inevitable to develop a mild reaction system with green solvents and reusable heterogeneous catalysts. Herein, we developed a one-pot tandem alcoholysis–hydrogenation route to transform polylactic acid into 1,2-PDO, in which ethanol was used as green solvent [33], and Raney Co, an efficient heterogeneous catalyst for hydrogenation of biomass [34], was employed for the catalysis reaction.

## 2. Materials and Methods

### 2.1. Materials

PLA powder (100 mesh) was purchased from Huachuang Plastic, Dongguan, China. PLA particle (Mw ~80,000) was purchased from Shanghai Macklin Biochemical Technology Co., Ltd. (Shanghai, China). PLA straws were obtained by Ningbo Sizhuo Plastic Industry Co., Ltd., Ningbo, China, and were shredded into particles with size < 5 mm before use. Standard substances (1,2-propanediol, ethyl lactate, and ethyl propionate, purity ≥ 99.5%), Raney Co (50 μm, dispersed in water), and internal standard substance (dodecane, purity of 99.5%) were obtained from Shanghai Aladdin Industrial Development Co., Ltd. (Shanghai, China). Other chemicals (analytical purity) were purchased from Sinopharm Reagent Co., Ltd. (Beijing, China).

### 2.2. Typical Alcoholysis and Hydrogenation Process

Typically, 1 g PLA powder, 10 mL ethanol, 0.1 g Raney Co, and 0.1 g dodecane were charged into a 50 mL Hastelloy batch autoclave. The vessel was sealed, and the mixture was flushed with H_2_ at least 5 times to remove air. Afterward, the reactor was purged with hydrogen to pre-set pressure (e.g., 3 MPa). The reactor was heated, and the reaction time was marked once the desired temperature was reached. After reaction, products were sampled and subjected to analysis. Moreover, methanolization of PLA straws without catalyst and hydrogen was carried out to determine the content of PLA.

### 2.3. Analysis

The reactants identification was carried out by Shimadzu QP 2010 Plus gas chromatography-mass spectrometry (GC-MS) with an Rtx-5MS column (30.0 m × 0.25 mm × 0.25 μm). The oven temperature was held at 60 °C for 5 min, and ramped to 260 °C for another 5 min at 12 °C/min. The injector and detector temperature were set at 280 °C and 285 °C, respectively.

The content of 1,2-propanediol (mol/L) was determined by a gas chromatograph (GC, Shimazu GC-2014C, Shimazu, Kyoto, Japan) equipped with a flame ionization detector (FID) and WondaCap FFAP capillary column (30.0 m × 0.25 mm × 0.25 μm). The initial column temperature was 60 °C holding for 5 min, and ramped to 260 °C at a rate of 12 °C/min. Both the injector and the detector temperature were set at 280 °C. The quantification was analyzed by the standard curves of each standard substance, using dodecane as the internal standard. The yield (%) of 1,2-PDO in the reaction bulk was calculated as Equation (1).

(1) $$\mathrm{Yield}\;\left(\%\right)=\frac{m_{i}}{m_{0}}\;\times \;100\%$$

where mi (g) is the mass quantity of 1,2-PDO and m_0_ (g) refers to the mass of the PLA material.

The conversion (%) of PLA feedstock was calculated as Equation (2).

(2) $$\mathrm{Conversion}\;\left(\%\right)=\frac{\left(m_{0}-m_{r}\right)}{m_{0}}\;\times \;100\%$$

where m_r_ (g) is the mass quantity of residues and m_0_ (g) refers to the mass of the PLA material.

### 2.4. Digestion PLA in Ethanol

A total of 1 g PLA powder and 10 mL ethanol were mixed in a 15-mL stoppered test tube, and then subjected to a digestion apparatus. The mixture was heated to a set temperature (100–240 °C), and the dissolution status of PLA was recorded at different times.

### 2.5. Calcination of PLA Straws

A total of 1 g of PLA straw pieces was submitted to an air furnace and heated to 600 °C at 10 °C/min, and then kept for another 3 h. After roasting, the residual inorganic ash was collected and weighed.

### 2.6. Fourier Transform Infrared (FTIR) Spectroscopy Analysis

The infrared spectra of PLA materials and its residues were recorded using a Spectrum Two LiTA Spectrometer (PerkinElmer, Waltham, MA, USA) with LiTaO_3_ Detector in the range of 4000 to 400 cm^−1^ with the resolution of 1 cm^−1^. The samples were grounded, mixed with KBr, and pressed into pellets for analysis.

### 2.7. Box-Benhken Optimization Design

Response surface methodology (RSM) was used to optimize experimental factors during the depolymerization process of PLA to recover 1,2-propanediol and investigate the correlation between the response and parameters. In this study, Box–Behnken design (BBD) was adopted as fewer experiment groups are needed to build a model equation than central composite design (CCD) [35]. A three-factor and three-level BBD was employed, requiring 17 runs (Table S2) to optimize the independent variables, reaction time (A, h), temperature (B, °C), and ethanol/PLA ratio (C, mL/g), using a Design Expert 11.0.4 software (Stat-Ease, Minneapolis, MN, USA).

## 3. Results and Discussion

### 3.1. Analysis of Alcoholysis and Hydrogenation Products

**Figure 1:**
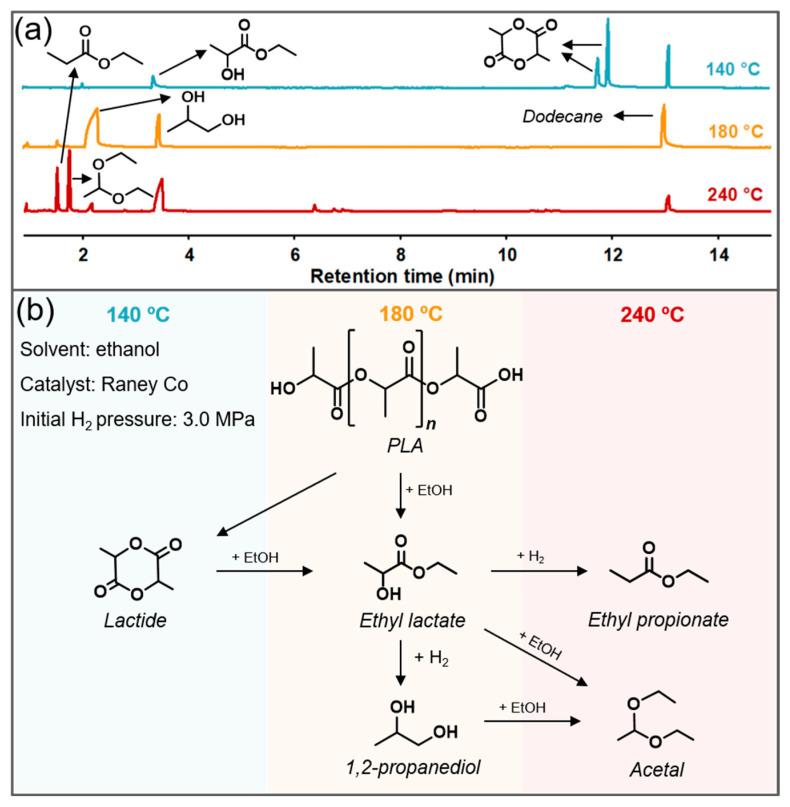
GC-MS analysis (**a**) and proposed mechanism (**b**) for tandem alcoholysis–hydrogenation of PLA. Reaction conditions: 1 g PLA, 10 mL ethanol, 0.1 g Raney Co, 3 MPa H_2_, 140–240 °C, and reacting for 12 h.

During the tandem alcoholysis–hydrogenation process of PLA, ethanol was employed both as nucleophile and solvent. Nucleophile attacks the carbonyl group in PLA macromolecule, leading to its alcoholytic depolymerisation with the formation of lactate esters [14]. Afterwards, residual ethanol can be used as reaction medium, and ethyl lactate as bio-based solution for further dissolving PLA [33,36]. Figure 1a shows the distribution of PLA powder-derived products, in which 1,2-propanediol was the primary product, along with ethyl lactate and negligible ethyl propionate as byproducts. In this reaction, toxic or ambiguity solvents were avoided, such as 1,4-dioxane [31], THF [30], and anisole [19], despite their solubility to PLA obviously stimulating the formation of 1,2-PDO. Meanwhile, methanol was also applied in this reaction system as it is a better nucleophile [37,38], and methyl lactate was the dominant product (Figure S1). Thence, ethanol is more optional in our alcoholysis–hydrogenation process owing to its safety and recyclability. Additionally, ethanol could be reused although it was involved in the reaction, and residual ethyl lactate could be further hydrogenated into 1,2-PDO, which catered to circular economy and eco-production.

Here, Raney Co was necessary in alcoholysis and hydrogenation of PLA to produce 1,2-PDO, and the proposed pathway is exhibited in Figure 1b. Under proper temperature (180 °C), ethyl lactate was evidenced to be the important intermediate during the degradation process, as it was the unique product of ethanolization for PLA without catalyst (Figure S2). Subsequently, ethyl lactate was transformed into 1,2-propanediol via hydrogenation in the presence of Raney Co and H_2_. In addition to Co-derived catalysts [39,40], Cu- [41,42] and Ru-based [27,43] catalysts have also been studied in catalyzing the hydrogenation of ethyl lactate to 1,2-PDO, but the low activity of Cu and the exorbitant price of Ru makes non-noble Co-derived catalysts more preferable in industrial application. As Figure 1a shows, inadequate alcoholysis and other side reactions occurred under low temperature and high temperature conditions, respectively. In the 140 °C run, PLA tended to degrade into dimer, different configurations of lactide, rather than alcoholytic depolymerisation into ethyl lactate. As feedstock, lactide would yield 1,2-PDO via tandem alcoholysis and hydrogenation process under suitable conditions. While at elevated temperature, ethyl propionate was more likely to be formed through the hydrogenation of -OH in ethyl lactate. Moreover, it was notable that a certain amount of acetal was available in degradation products, which might be the coupling product of decarboxylative acceptorless dehydrogenation of ethyl lactate [44].

### 3.2. Effect of Single Factor

**Figure 2:**
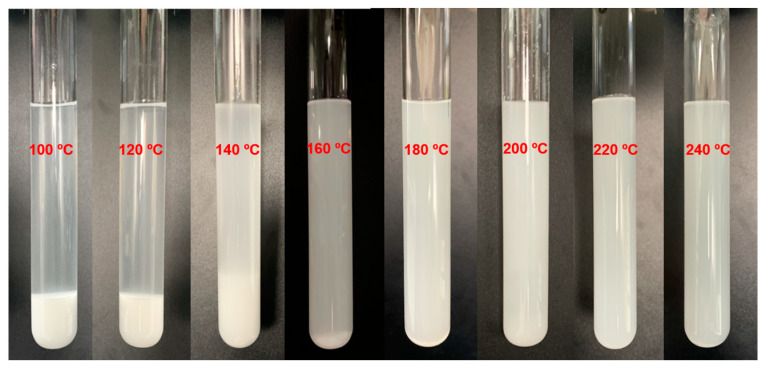
Digestion of PLA powder in ethanol. Reaction conditions: 1 g PLA powder, 10 mL ethanol, andheating to 100, 120, 140, 160, 180, and 200 °C for 10 min.

**Figure 3:**
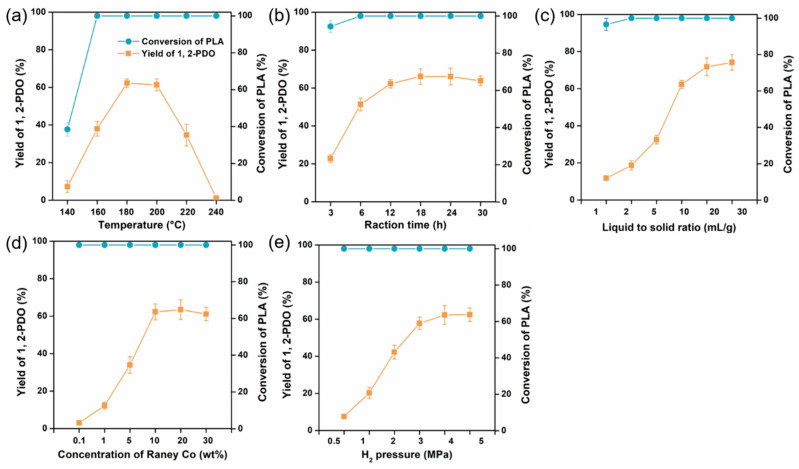
Effect of different parameters: (**a**) temperature, (**b**) reaction time, (**c**) liquid to solid ratio, (**d**) concentration of Raney Co, and (**e**) H_2_ pressure on the yield of 1,2-PDO and conversion of PLA.

To scan the effect of different parameters on the yield of 1,2-propanediol from the alcoholysis and hydrogenation of PLA, single-factor tests were carried out. Due to the insolubility of PLA powder in ethanol, the degradation temperature was scanned by a digestion test of PLA in ethanol (Figure 2). When the temperature was below 120 °C, the PLA powder was almost deposited in the bottom of tube and the solvent was kept as transparent. Further increasing the digestion temperature from 140 to 160 °C, the PLA powder was gradually dissolved and dispersed in ethanol, and total digestion was accomplished at 180 °C. Considering the reaction adequacy, a temperature range of 140–240 °C was investigated towards the effect on PLA conversion and 1,2-PDO yield in Figure 3a. As the data showed, temperature violently affected the conversion of raw material and the composition of products. Under relatively low temperature (140–160 °C), total conversion of PLA could not be achieved, and PLA was more easily to form lactide rather than ethyl lactate, through back-biting depolymerization or radical process [45,46] (Figure 1a). However, elevated temperature (220–240 °C) favored to form more byproducts, such as ethyl propionate and acetal, which resulted in barely any formation of 1,2-propanediol.

Sufficient reaction time was necessary to completely degrade PLA and promote the formation of 1,2-propanediol. Figure 3b demonstrates that 6 h was required for 100% conversion of PLA, and the longer reaction time still was needed to gain higher yield of 1,2-PDO. However, further increasing the reaction time (>18 h) even led to a slight decrease in the yield of target product. Meanwhile, adequate ethanol dosage (≥2 mL/g PLA) was also indispensable for full conversion, and larger amount of ethanol (10–30 mL /g PLA) was necessary to yield 1,2-PDO (Figure 3c). Considering cost and energy consumption, the range of 160–200 °C, 6–18 h, and 10–30 mL ethanol/g PLA was selected for further optimization.

In addition, another two parameters were investigated to select a suitable value of Raney Co dosage and H_2_ pressure (Figure 3d,e). Figure S2 indicated that Raney Co and hydrogen were integral for the formation of 1,2-PDO, and ethyl lactate was the main product in the absence of Raney Co or H_2_ or both. The yield of 1,2-PDO increased sharply along with the increase in catalyst dosage and H_2_ pressure at initial stage, and then tended to invariability. Thus, 10 wt% Raney Co and 3 MPa H_2_ were used in the following tests.

### 3.3. Optimization of Reaction Procedure

A three-factor and three-level BBD consisting of 17 experimental runs was used to optimize the tandem alcoholysis–hydrogenation process of PLA. Table S2 summarizes the experimental yield of 1,2-propanediol and predicted values by model, which was fitted as a quadratic polynomial equation shown as Equation (3):

Yield (1,2-propanediol, %) = 69.3 + 5.1 A + 13.7 B + 5.3 C + 0.6 AB + 2.1 AC −  0.2 BC − 4.3 A^2^ − 18.0 B^2^ − 4.0 C^2^ (3)

where A, B, and C are the coded value of reaction time, temperature, and ethanol/PLA ratio, respectively. The sign of coefficients implied how the parameters influence the response. Therein, positive coefficients represent related factors have synergistic effect, but otherwise factors have antagonist effect towards response [45].

Statistical testing of regression model was checked by F-test, and the analysis of variance (ANOVA) for the fitted quadratic polynomial equation is shown in Table S3. Additionally, the model was significant as the F-value was 56.7, while the lack of fit F-value of 2.6 indicated no statistical significance relative to the pure error as the *p*-value is higher than 0.05, which is desirable as the model exhibits good fitting to the relative response [47]. Moreover, the validation of the polynomial regression model was confirmed by the comparative plot between experimental and predicted values in Figure S3a, in which the coefficient (R^2^ = 0.99) indicated that the fitted model was well in agreement with actual results [48]. In addition, the normal plot residuals in Figure S3b displayed the normal probability and studentized residuals lie reasonably in a straight line, implying the significance of the fitted model, and confirming that the assumption of the analysis was satisfied [49]. 

**Figure 4:**
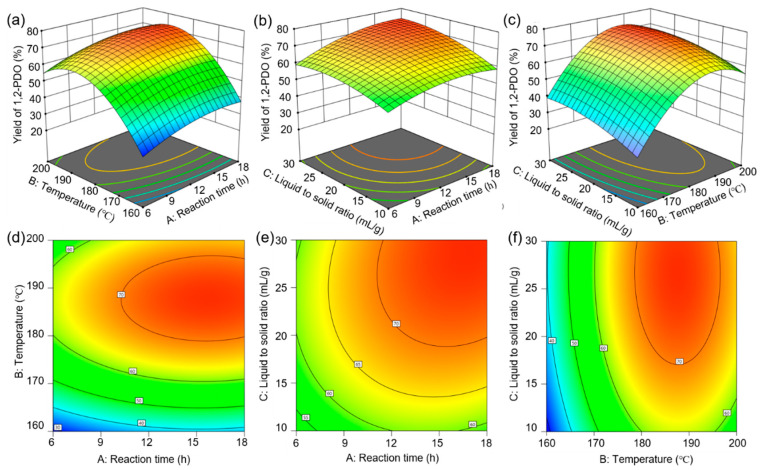
Response surface (**a**–**c**) and contour plots (**d**–**f**) for yield of 1,2-PDO.

The 3D response surface plots and 2D contour plots in Figure 4, i.e., the graphical and visualized representations of the polynomial regression model, could help to better understand the individual and interactions of the variables during the process of alcoholysis and hydrogenation of PLA. Additionally, the optimal reaction conditions were optimized by the 3D response surface with Box–Behnken design as follows: 187 °C of reaction temperature, 15.6 h of reaction time, and 27 mL ethanol/g PLA, which contributed to the predicted maximum yield of 1,2-PDO as 76.1%.

### 3.4. Verification for Optimal Model

**Figure 5:**
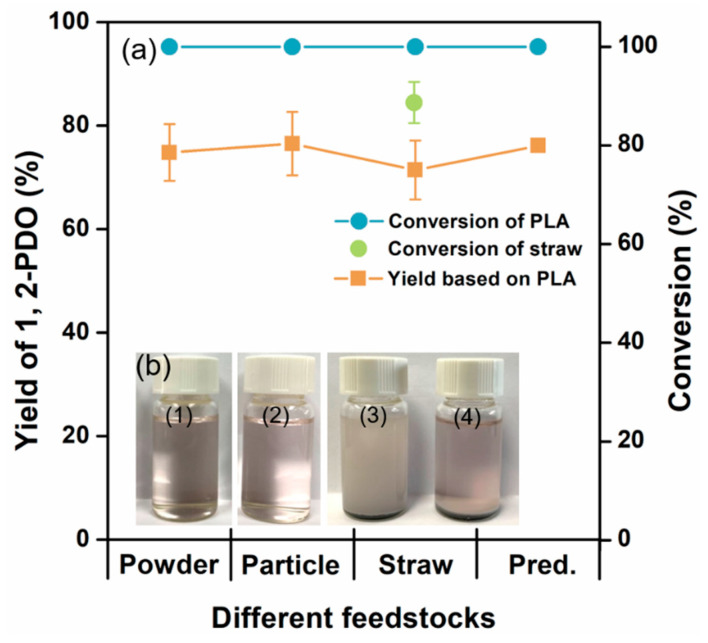
Verification experiments for optimal model. (**a**) Yield of 1,2-propanediol and conversion derived from different feedstocks vs. predicted (Pred.) values from the model. The recovery of 1,2-PDO was based on the mass of feedstock. (**b**) Depolymerized samples of PLA powder (1), particle (2), dispersed (3), and sedimented insoluble particles (4) of PLA-straws. Reaction conditions: 1 g feedstock, 3 MPa H_2_, 0.1 g Raney Co, 27 mL ethanol, 187 °C, and reacting for 15.6 h.

As discussed above, the model of tandem alcoholysis–hydrogenation of PLA to produce 1,2-PDO was established, and the predicted optimal conditions were optimized. To verify the reliability of the model, three PLA materials (PLA powder, PLA particle, and commercially available PLA-straws) were submitted to the reaction under the optimal conditions in Figure 5. Both PLA powder and particle were totally depolymerized, and 74.8 ± 5.5% and 76.5 ± 6.2% of 1,2-PDO were obtained, respectively, which indicated the applicability of the model as the difference between actual and predicted value was not significant (*p* > 0.05).

Under the same conditions, total conversion of PLA and 71.4 ± 5.7% yield of 1,2-PDO were achieved based on the mass of PLA in the straws, and the conversion of PLA straws was 88.9 ± 4.2%. As the FTIR spectra of neat PLA powder, reaction residues and roasting residues of PLA straws were compared in Figure S4, the PLA ingredient was totally converted as the specific peaks of PLA were absent in the spectra of residues. However, insoluble particles remained in the final samples (Figure 5b), which might be some inorganic additives (such as talcum [50], diatomite [51]) in PLA straws as there were 10.4% residues after roasting PLA straws at 600 °C. Moreover, some additional C4 derivatives were formed in PLA straws degradation reaction, such as butanediol, diethyl butanedioate, and ethyl hydroxybutyl butanedioate (Figure S5), which was supposed to be the organic agents (e.g., co-polymers: poly(butylene succinate) [52], poly(butylene adipate-co-terephthalate) [53], and biomass: lignin [54] and coffee ground [55]). These additives in PLA straws are used to modify its physical limitations, such as softening temperature, brittleness, slow crystallization, etc. [56]. Consequently, the model is also suitable for the depolymerization of PLA straws and other commercial products if the PLA content could be determined.

### 3.5. Reusability Test of Catalyst

**Figure 6:**
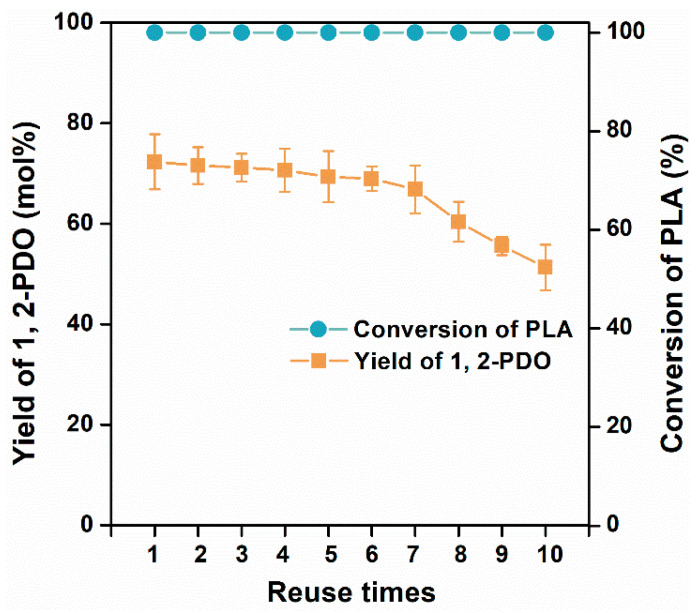
Reusability of Raney Co for alcoholysis and hydrogenation of PLA. Reaction conditions: 1 g PLA, 3 MPa H_2_, 0.1 g Raney Co, 27 mL ethanol, 187 °C, and reacting for 15.6 h.

Under the optimized condition, Raney Co was recycled and reused ten times, and the results are shown in Figure 6. Overall, total conversion of PLA powder was achieved for all runs, and the catalytic activity for alcoholysis–hydrogenation of PLA was maintained at least 7 times with only a 4.9% decrease in the yield of 1,2-propanediol. Continuing reusing the catalyst gradually led to an obvious decrease in catalytic properties, as there was a 29.1% reduction of 1,2-PDO yield in the tenth run. Thus, further effects should be focused on improving the selectivity and stability of Co-based catalysts.

## 4. Conclusions

In this work, a one-pot tandem alcoholysis and hydrogenation of PLA was successfully conducted to produce 1,2-PDO using Raney Co catalyst, and ethanol was used both as reagent for esterification as well as solvent for subsequent hydrogenation. The reaction conditions were optimized by single-factor analysis and response surface optimization as 15.9 h reaction time, 187 °C temperature, and 27 mL ethanol/g PLA, and 1,2-PDO yield of 74.8 ± 5.5%, 76.5 ± 6.2%, and 71.4 ± 5.7% were derived from powder, particle, and straws of PLA, respectively. The Raney Co catalyst was efficient on a gram scale, and can be reused in at least seven degradation cycles, suggesting the possibility of industrial application of this method. Finally, this work demonstrated that it is possible to recycle PLA wastes using inexpensive and commercially available catalyst, and eco-friendly and multi-functional solvent, contributing to reduce and digest the end-of-life PLA residues in the environment, which offers a sustainable alternative to the depolymerization of PLA wastes.

## Data Availability

The data that support the findings of this study are available on request from the corresponding author.

## Supplementary Materials

The following supporting information can be downloaded at: https://www.mdpi.com/article/10.3390/polym15020413/s1. Figure S1. GC-MS analysis of methanolization and hydrogenation products from PLA powder and methyl lactate; Figure S2. GC-MS analysis of controlled experiments; Figure S3. Validation of fitted model; Figure S4. FTIR spectra of PLA and its residues; Figure S5. GC-MS analysis of different PLA products-derived degradation liquids; Table S1. Bio-based processes examples towards 1,2-PDO production [18,19,24,27,30,31,32,43,57,58,59,60,61,62,63,64,65,66]; Table S2. Experimental design and observed response; Table S3. ANOVA results of the quadratic model.
