# Supplementary material for: One-Pot Tandem Alcoholysis-Hydrogenation of Polylactic Acid to 1,2-Propanediol
Source: Polymers (Basel). 2023 Jan 12;15(2):413. doi: 10.3390/polym15020413 (PMC9864359; doi:10.3390/polym15020413)
Supplement: Supplementary file 1 [file polymers-15-00413-s001.zip › polymers-2152843-supplementary.pdf]

polymers

Supplementary Information For

One-Pot Tandem Alcoholysis-Hydrogenation of Polylactic Acid to 1,2-Propanediol

Jialin Xu [1,2], Kuo Zhou [3], Linlin Qin [1], Zaiming Tan [1], Shijing Huang [1], Peigao Duan [2] and Shimin Kang [1,*]

[1] Engineering Research Center of None-Food Biomass Efficient Pyrolysis and Utilization Technology of Guangdong Higher Education Institutes, Guangdong Provincial Key Laboratory of Distributed Energy Systems, Dongguan University of Technology, Dongguan 523808, China
[2] School of Chemical Engineering and Technology, Xi'an Jiaotong University, Xi'an 710049, China
[3] Department of Chemistry, Lishui University, Lishui 323000, China
* Correspondence: kangshimin@dgut.edu.cn

Contents

1. Figures

1.1 **Figure S1.** GC-MS analysis of methanolization and hydrogenation products from PLA powder and methyl lactate.

1.2 **Figure S2.** GC-MS analysis of controlled experiments.

1.3 **Figure S3.** Validation of fitted model.

1.4 **Figure S4.** FTIR spectra of PLA and its residues.

1.5 **Figure S5.** GC-MS analysis of different PLA products-derived degradation liquids.

2. Tables

2.1 **Table S1.** Bio-based processes examples towards 1,2-PDO production.

2.2 **Table S2.** Experimental design and observed response.

2.3 **Table S3.** ANOVA results of the quadratic model.

3. Reference

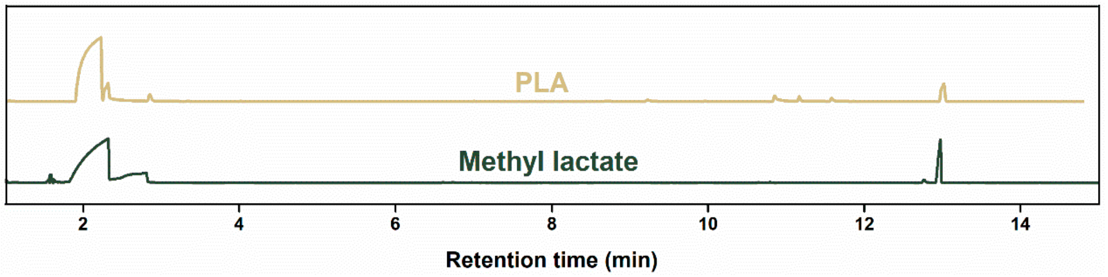

Figure S1. GC-MS analysis of methanolization and hydrogenation products from PLA powder and methyl lactate.

Reaction conditions: 1 g feedstock, 10 mL methanol, 3.0 MPa H_2, 0.1 g Raney Co, reacting for 12 h.

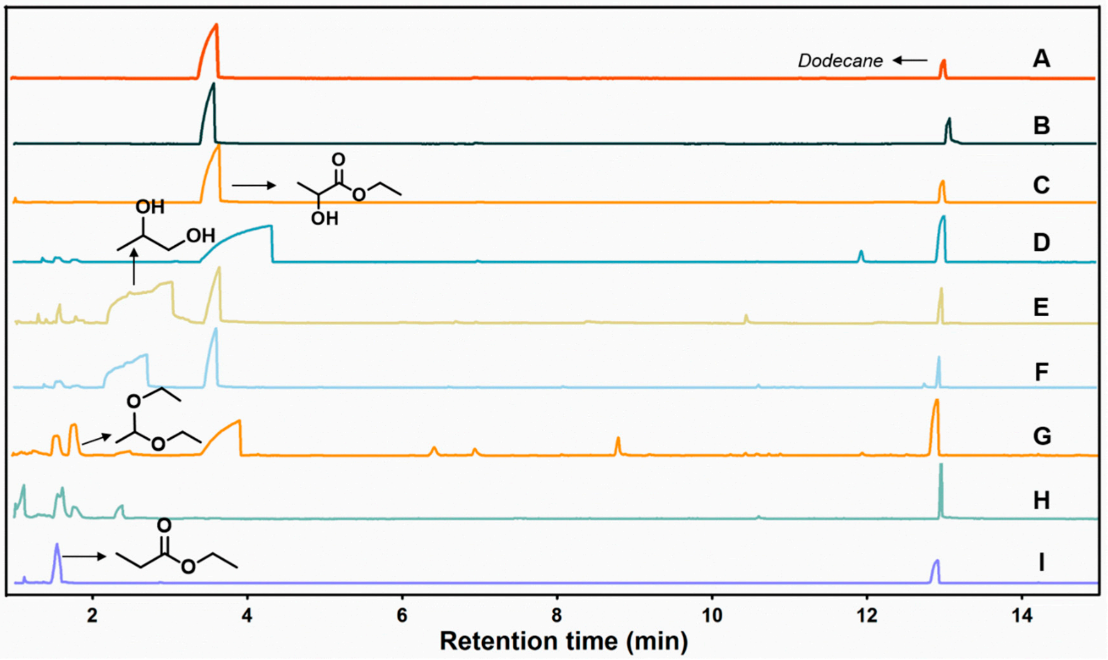

Figure S2. GC-MS analysis of control experiments. (A) 1 g PLA powder, 10 mL ethanol, 0.3 MPa N_2, 180 ºC, (B) 1 g PLA powder, 10 mL ethanol, 3 MPa H_2, 180 ºC, (C) 1 g PLA powder, 10 mL ethanol, 0.3 MPa N_2, 0.1 g Raney Co, 180 ºC, (D) 1 g lactide, 10 mL ethanol, 0.3 MPa N_2, 180 ºC, (E) 1 g lactide, 10 mL ethanol, 3 MPa H_2, Raney Co, 180 ºC, (F) 1 g ethyl lactate, 10 mL ethanol, 3 MPa H_2, Raney Co, 180 ºC, (G) 1 g ethyl lactate, 10 mL ethanol, 3 MPa H_2, Raney Co, 240 ºC, (H) 1 g 1,2-propanediol, 10 mL ethanol, 3 MPa H_2, Raney Co, 240 ºC, (I) 1 g ethyl propionate, 10 mL ethanol, 3 MPa H_2, Raney Co, 240 ºC.

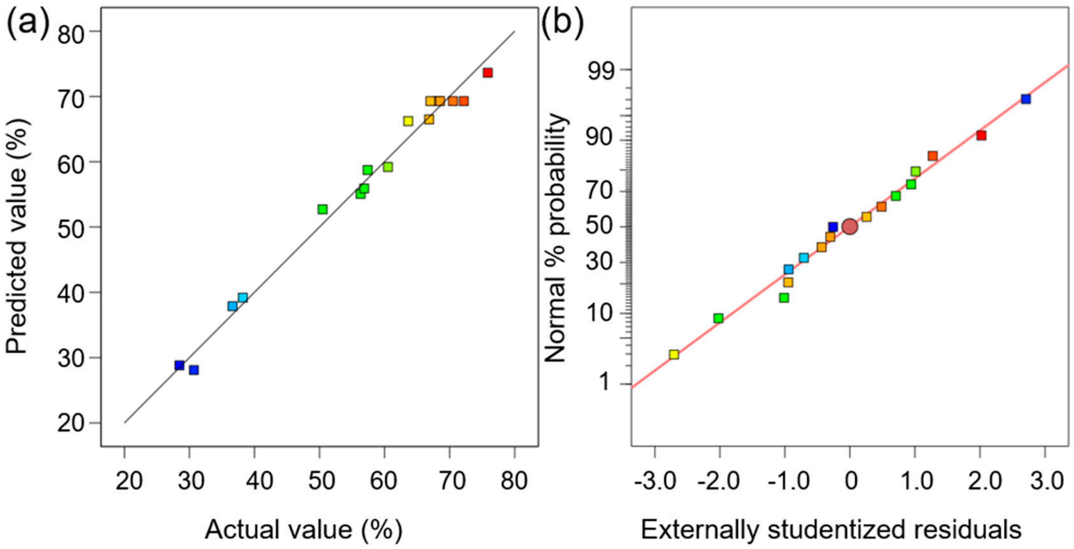

Figure S3. Validation of fitted model. (a) Comparative plot between actual and predicted values; (b) normal plot of residuals between normal % probability versus studentized residuals.

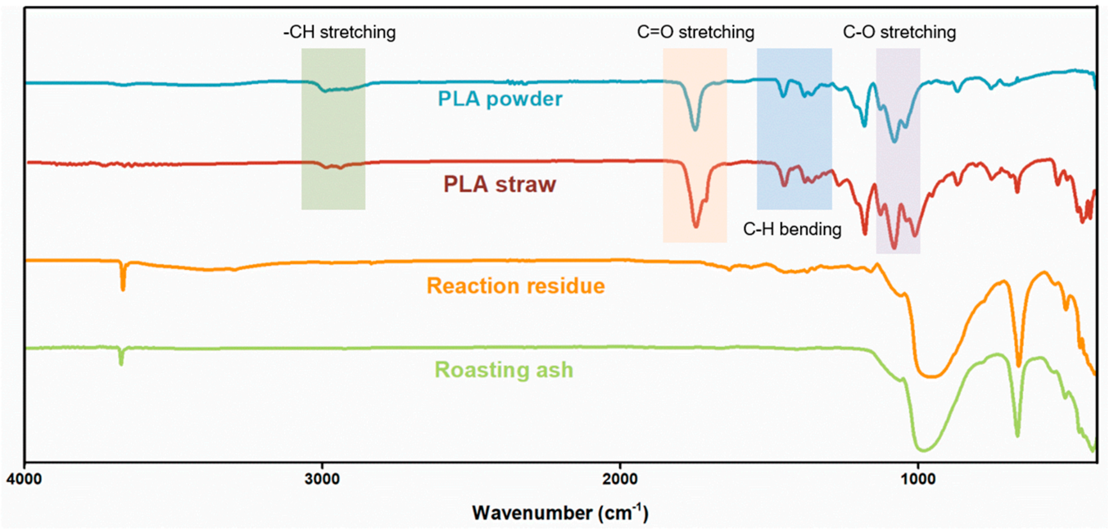

Figure S4. FTIR spectra of PLA and its residues. Residues formation conditions: 1 g PLA straws, 3 MPa H_2, 0.1 g Raney Co, 27 mL ethanol, 187 °C, and reacting for 15.6 h.

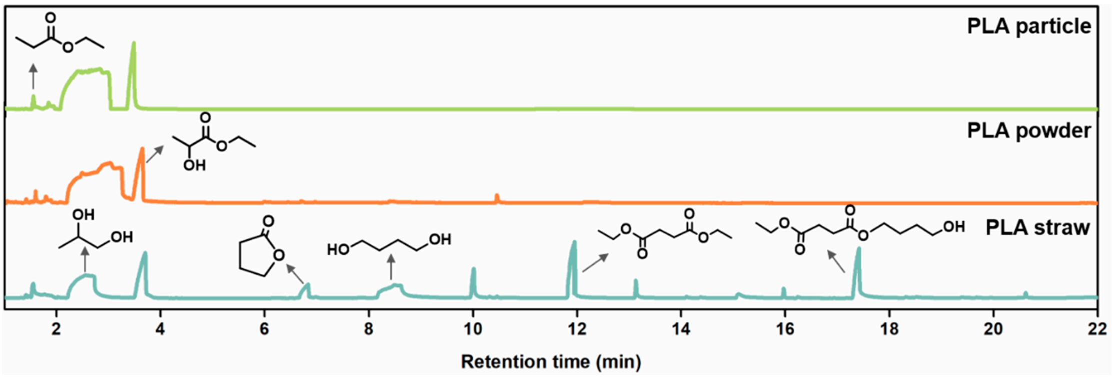

Figure S5. GC-MS analysis of different PLA products-derived degradation liquids. Reaction conditions: 1 g feedstock, 3 MPa H_2, 0.1 g Raney Co, 27 mL ethanol, 187 °C, and reacting for 15.6 h.

Table S1. Bio-based processes examples towards 1,2-PDO production.

Feedstock	Solvent	Catalyst	Reaction conditions			Conversion (%)	Selectivity (%)	Reference
			Temp. (°C)	H_2 Press. (Bar)	*T* (h)			
Glycerol	H_2O	Cu/MgO	210	45	12	96	93	[1]
Glycerol	-	Pd-CuCr	220	60	12	83	93	[2]
Glycerol	EtOH	Rh-Cu	180	20	10	91	99	[3]
Glycerol	H_2O	Cu/Mg/Al	180	30	20	91	96	[4]
Glycerol	H_2O	CuPd/TiO_2-Na	220	7	6	65	85	[5]
Cellulose	H_2O	Ni/ZnO	245	60	2	>99	34.4	[6]
Glucose	H_2O	CuCrCa	140	60	2	>99	53	[7]
Lactic acid	H_2O	Ru/C	120	35	2.5	23	>99	[8]
Lactic acid	H_2O	Cu/SiO_2	200	7.5	-	>99	88	[9]
Ethyl lactate	Heptane	Ru-B/Al_2O_3	150	55	10	91	92	[10]
Ethyl lactate	Heptane	CoB	160	60	9	98	>99	[11]
Ethyl lactate	H_2O	Ru/SiO_2	160	50	8	83	92	[12]
Lactide	MeOH	Cu/Cr/Ba	150	150	15	>99	>99	[13]
Lactide	THF	Ru(II) PNN	110	10	48	>99	87	[14]
PLA	MeOH	Cu/Cr/Ba	150	150	15	>99	88	[13]
PLA	Toluene	$Zn(OAc)_2·2H_2O$	110	$(EtO)_2MeSiH$	48	71	92	[15]
PLA	THF	Ru-MACHO-BH	140	45	3	>99	>99	[16]
PLA	1,4-Dioxane	Ru(triphos)tmm, $HNTf_2$	140	100	16	>99	>99	[17]
PLA	Anisole/THF	Ru(II) PNN	160	54.4	48	>99	-	[18]

Table S2. Experimental design and observed response.

Variables	-1	0	1
Time (h)	6	12	18
Temperature (°C)	160	180	200
Ethanol/PLA (mL/g)	10	20	30

Run	Coded levels			Yield of 1,2-propanediol (%)	
	A	B	C	Experimental (mean ± SD)	Predicted
1	-1	0	1	60.5 ± 2.3	59.2
2	0	-1	1	38.2 ± 1.8	39.2
3	-1	0	-1	50.5 ± 0.7	52.7
4	0	0	0	70.5 ± 4.1	69.3
5	0	1	1	63.6 ± 3.0	66.2

6	1	0	1	75.8 ± 4.5	73.6
7	1	1	0	66.9 ± 3.5	66.5
8	0	0	0	68.2 ± 2.1	69.3
9	0	0	0	72.2 ± 5.1	69.3
10	0	0	0	68.5 ± 3.3	69.3
11	0	0	0	67.0 ± 4.7	69.3
12	0	1	-1	56.9 ± 3.4	55.9
13	-1	-1	0	28.5 ± 1.8	28.8
14	1	0	-1	57.4 ± 5.6	58.7
15	-1	1	0	56.3 ± 1.1	55.1
16	0	-1	-1	30.7 ± 2.2	28.1
17	1	-1	0	36.6 ± 2.9	37.9

Source	Sum of Aquares	df	F value	p-value	
Model	3549.8	9	56.7	< 0.0001	significant
A	209.4	1	30.2	0.0009	
B	1503.9	1	216.3	< 0.0001	
C	228.9	1	32.9	0.0007	
AB	1.39	1	0.2	0.7	
AC	17.77	1	2.6	0.2	
BC	0.15	1	0.02	0.9	
A^2	76.2	1	11.0	0.01	
B^2	1359.4	1	195.5	< 0.0001	
C^2	66.3	1	9.5	0.02	
Residual	48.7	7			
Lack of fit	32.1	3	2.6	0.2	not significant
Pure Error	16.6	4			
Cor Total	3598.5	16			
R^2 = 0.99, Adj R^2 = 0.97, Pred. R^2 = 0.85, Adep Precision = 22.52, C.V.% = 4.63					

Table S3. ANOVA results of the quadratic model.

Reference

1. Pandhare, N.; Pudi, S.M.; Mondal, S.; Pareta, K.; Kumar, M.; Biswas, P. Development of Kinetic Model for Hydrogenolysis of Glycerol over Cu/MgO Catalyst in a Slurry Reactor. *Ind. Eng. Chem. Res.* **2017**, *57*, 101–110, https://doi.org/10.1021/acs.iecr.7b03684.
2. Kim, N.D.; Park, J.R.; Park, D.S.; Kwak, B.K.; Yi, J. Promoter effect of Pd in CuCr2O4 catalysts on the hydrogenolysis of glycerol to 1,2-propanediol. *Green Chem.* **2012**, *14*, 2638–2646, https://doi.org/10.1039/c2gc00009a.
3. Xia, S.; Yuan, Z.; Wang, L.; Chen, P.; Hou, Z. Catalytic production of 1,2-propanediol from glycerol in bio-ethanol solvent. *Bioresour. Technol.* **2012**, *104*, 814–817, https://doi.org/10.1016/j.biortech.2011.11.031.
4. Yuan, Z.; Wang, L.; Wang, J.; Xia, S.; Chen, P.; Hou, Z.; Zheng, X. Hydrogenolysis of glycerol over homogenously dispersed copper on solid base catalysts. *Appl. Catal. B: Environ.* **2011**, *101*, 431–440, https://doi.org/10.1016/j.apcatb.2010.10.013.
5. Ardila, A.N.; Sánchez-Castillo, M.A.; Zepeda, T.A.; Villa, A.L.; Fuentes, G.A. Glycerol hydrodeoxygenation to 1,2-propanediol catalyzed by CuPd/TiO2-Na. *Appl. Catal. B: Environ.* **2017**, *219*, 658–671, https://doi.org/10.1016/j.apcatb.2017.08.006.
6. Wang, X.; Meng, L.; Wu, F.; Jiang, Y.; Wang, L.; Mu, X. Efficient conversion of microcrystalline cellulose to 1,2-alkanediols over supported Ni catalysts. *Green Chem.* **2012**, *14*, 758–765, https://doi.org/10.1039/c2gc15946e.
7. Xiao, Z.; Jin, S.; Sha, G.; Williams, C.T.; Liang, C. Two-Step Conversion of Biomass-Derived Glucose with High Concentration over Cu–Cr Catalysts. *Ind. Eng. Chem. Res.* **2014**, *53*, 8735–8743, https://doi.org/10.1021/ie5012189.
8. Iqbal, S.; Kondrat, S.A.; Jones, D.R.; Schoenmakers, D.C.; Edwards, J.K.; Lu, L.; Yeo, B.R.; Wells, P.P.; Gibson, E.K.; Morgan, D.J.; et al. Ruthenium Nanoparticles Supported on Carbon: An Active Catalyst for the Hydrogenation of Lactic Acid to 1,2-Propanediol. *ACS Catal.* **2015**, *5*, 5047–5059, https://doi.org/10.1021/acscatal.5b00625.
9. Cortright, R.; Sanchez-Castillo, M.; Dumesic, J. Conversion of biomass to 1,2-propanediol by selective catalytic hydrogenation of lactic acid over silica-supported copper. *Appl. Catal. B: Environ.* **2002**, *39*, 353–359, https://doi.org/10.1016/s0926-3373(02)00121-2.
10. Luo, G., Yan, S., Qiao, M., Zhuang, J., Fan, K. Effect of tin on Ru-B/γ-Al2O3 catalyst for the hydrogenation of ethyl lactate to 1, 2-propanediol. *Appl. Catal. A: Gen.* **2004**, *275*, 95–102.
11. Ma, X.; Sun, D.; Zhao, F.; Du, C. Liquid phase hydrogenation of biomass-derived ethyl lactate to propane-1,2-diol over a highly active CoB amorphous catalyst. *Catal. Commun.* **2015**, *60*, 124–128, https://doi.org/10.1016/j.catcom.2014.11.027.
12. Feng, J.; Xiong, W.; Jia, Y.; Wang, J.; Liu, D.; Qin, R. Hydrogenation of ethyl lactate over ruthenium catalysts in an additive-free catalytic system. *React. Kinet. Catal. Lett.* **2011**, *104*, 89–97, https://doi.org/10.1007/s11144-011-0329-9.
13. Shuklov, I.A.; Dubrovina, N.V.; Schulze, J.; Tietz, W.; Kühlein, K.; Börner, A. Propane-1,2-diols from Dilactides, Oligolactides, or Poly-L-lactic Acid (PLLA): From Plastic Waste to Chiral Bulk Chemicals. *Chem. – A Eur. J.* **2014**, *20*, 957–960, https://doi.org/10.1002/chem.201302004.
14. Balaraman, E.; Fogler, E.; Milstein, D. Efficient hydrogenation of biomass-derived cyclic di-esters to 1,2-diols. *Chem. Commun.* **2011**, *48*, 1111–1113, https://doi.org/10.1039/c1cc15778g.
15. Fernandes, A.C., Reductive depolymerization of plastic waste catalyzed by $Zn(OAc)_2{\cdot}2H_2O$. *ChemSusChem*, **2021**, *14*, 4228–4233.
16. Kindler, T.O., Alberti, C., Fedorenko, E., Santangelo, N., Enthaler, S., Ruthenium-catalyzed hydrogenative degradation of end-of-life poly(lactide) to produce 1,2-propanediol as platform chemical. *ChemistryOpen* **2020**, *9*, 401–404.
17. Westhues, S.; Idel, J.; Klankermayer, J. Molecular catalyst systems as key enablers for tailored polyesters and polycarbonate recycling concepts. *Sci. Adv.* **2018**, *4*, eaat9669, https://doi.org/10.1126/sciadv.aat9669.
18. Krall, E.M.; Klein, T.W.; Andersen, R.J.; Nett, A.J.; Glasgow, R.W.; Reader, D.S.; Dauphinais, B.C.; Mc Ilrath, S.P.; Fischer, A.A.; Carney, M.J.; et al. Controlled hydrogenative depolymerization of polyesters and polycarbonates catalyzed by ruthenium(ii) PNN pincer complexes. *Chem. Commun.* **2014**, *50*, 4884–4887, https://doi.org/10.1039/c4cc00541d.
